# May Patients with Recurrent Venous Disease Benefit from Sequential Treatment More than Those without Previous Intervention? A Single-Center Retrospective Study on the Safety and Efficacy of Abdominal and Pelvic Veins Embolization in Sequential Approach

**DOI:** 10.3390/jcm13175053

**Published:** 2024-08-26

**Authors:** Cezary Szary, Justyna Wilczko-Kucharska, Krzysztof Celejewski, Małgorzata Łodyga, Marcin Napierala, Dominika Plucinska, Siavash Swieczkowski-Feiz, Jerzy Leszczynski, Michal Zawadzki, Tomasz Grzela

**Affiliations:** 1 Clinic of Phlebology, 02-034 Warsaw, Poland; 2 Department of General, Endocrinological and Vascular Surgery, Central University Hospital, Medical University of Warsaw, 02-091 Warsaw, Poland; 3 Department of Congenital Heart Diseases, National Institute of Cardiology, 04-628 Warsaw, Poland; 4 Maria Sklodowska-Curie National Research Institute of Oncology, 02-781 Warsaw, Poland; 5 Center of Postgraduate Medical Education, 01-813 Warsaw, Poland; 6 Center of Biostructure Research, Medical University of Warsaw, 02-004 Warsaw, Poland

**Keywords:** abdominal/pelvic veins, embolization, compartment, recurrent venous disease, safety, sequential treatment, symptoms change

## Abstract

**Background/Objective:** The endovenous embolization of insufficient abdominal/pelvic veins is the preferred method of treatment. Also, it seems to be crucial in the treatment of lower limb vein insufficiency, particularly in recurrent disease. This study aimed to evaluate of pelvic vein embolization safety and its impact on the short-term outcome in the sequential treatment of venous disease. **Methods:** A retrospective analysis involved data from 506 female patients with venous disease involving abdominal and pelvic veins. All records were extracted from the medical database and included patient history, imaging reports as well as pre- and post-operative surveys. **Results:** Among the patients analyzed, 37.2% underwent some venous intervention in the past, with significant differences in symptom severity between groups. The embolization procedure revealed a high safety profile, with no serious complications. Pain during and after the procedure was generally low, with significantly lower pain scores in patients with recurrence. In patients who required left renal vein venoplasty a 1.7-fold increased risk of lumbar pain after embolization and venoplasty procedure was observed. Overall, 66.6% of patients reported improvement in pelvic symptoms and 72.1% experienced improvement in leg symptoms. The full sequential treatment protocol (abdominal, pelvic, and leg compartment) demonstrated superior outcomes in leg symptom improvement compared to embolization alone. **Conclusions:** Pelvic vein embolization is a safe and effective method of treatment, significantly improving both pelvic and leg symptoms, particularly in patients with a history of previous interventions in lower limb veins. Further studies are warranted to validate our findings and further refine treatment protocols.

## 1. Introduction

The endovenous embolization (EMBO) of pelvic and abdominal veins is currently the first-choice method in the treatment of their insufficiency [1,2,3,4]. However, recent studies provide strong evidence that, at least in some patients, the EMBO procedure may also be useful as a part of the treatment for lower-limb venous insufficiency (LLVI) [5,6]. Several reports support the thesis that pelvic vein insufficiency (PVI) seems to be associated with the occurrence and progression of venous disease in the lower limb compartment, whereas the pelvic origin of LLVI should be suspected mainly in multiparous women, patients with recurrent venous disease (ReVD), and in young patients with early onset of venous insufficiency [7,8,9].

This relationship led to the redefinition of venous disease as a more complex problem, not limited only to the lower limb compartment, and consequently, it required a thorough change in the strategy of venous treatment. According to that strategy, the EMBO procedure has become a key component of the sequential treatment of venous disease, where it usually should precede the intervention in the lower limb compartment [5,6].

Although the involvement of the EMBO procedure in sequential treatment follows the recommendations of the European Society for Vascular Surgery [10] and recent Guidelines of the International Union of Phlebology [1], it still attracts some criticism, mainly among the most conservative phlebology experts and some vascular surgeons [11,12]. The main controversy concerns the indication for EMBO treatment in patients with significant changes in both pelvic and lower limb veins but with relatively low symptoms in the abdominal/pelvic compartment and more pronounced in the legs. The clinicians skeptical of the sequential treatment argue that in such cases, the EMBO procedure is not necessary and the intervention should be limited to the lower limb compartment only [10,12]. However, our recent study as well as some other reports clearly show that the issue may be the wrong interpretation of symptom distribution [6,13]. High intensity of symptoms in legs with weak pain in the pelvic compartment does not necessarily mean that only the lower limb veins are affected, since the lower limb compartment may act as a kind of retention buffer for overloaded pelvic veins. Not only the high frequency of PVI in patients with ReVD [9] supports this hypothesis but also the observation that in the sequential hemodynamic treatment, the embolization of abdominal and pelvic veins resulted in a significant reduction of overload in the lower limb compartment. In our study, we have found that in nearly 20% of patients initially qualified for the LLVI treatment, the EMBO led to the reduction of reflux in leg superficial veins below the clinically relevant limit of 0.5 s and these patients did not require any further intervention [6].

Apart from a poor understanding of the aforementioned sequential treatment strategy, its criticism or refusal may also result from doubts in regard to the efficacy and safety of the EMBO procedure, including patients’ exposure to X-rays or possible severe and delayed complications [14,15]. Indisputably, the current literature is still limited mainly to case series, with a lack of data from randomized trials [1,2,4,14,16]. Furthermore, the relatively low number of specialized centers experienced in the EMBO procedures does not improve this situation. Another interesting issue is the safety and effectiveness of the EMBO procedure in the sequential treatment of patients with ReVD, after previous unsuccessful leg treatment, especially since those patients constitute 25–40% of the population with venous disease, treated in many phlebology centers [6,9].

Therefore, the aim of our study was the retrospective analysis of single center experience with the pelvic vein embolization procedure in regards to its safety and short-term outcome assessed in symptoms intensity change surveys. Furthermore, the evaluation concerned the possible impact of the previous leg treatment on both the safety and efficacy of the EMBO procedure alone or as a component of the sequential venous treatment.

## 2. Materials and Methods

The study concerned previously collected data of patients with pelvic venous disease who underwent routine diagnostics and treatment in the Clinic of Phlebology during the 2020-2023 period. The medical database of the clinic was searched using specified inclusion criteria: venous disease involving the abdominal and pelvic veins; age > 18 years; date range for the embolization procedure—from January 2020 to November 2023. Furthermore, to avoid sex-related data variability, only females were included in the analysis. Data of patients with thromboembolism, advanced endometriosis, any active neoplasm, diagnosed inflammatory bowel disease, or any other unstable chronic disease that would disqualify the patient from the endovascular procedure were not included in the assessment.

Although any formal approval is not required due to the retrospective character of the study, the concept of this project was submitted to the Local Ethics Committee at the Medical University of Warsaw (statement No. AKBE/181/2020).

The individual patients’ records containing all available data were extracted, anonymized, and then subjected to further statistical evaluation. All data were collected during routine diagnostic and treatment procedures, according to the standard protocol of our clinic, as previously described [8,17]. They included medical history, especially previous vein treatment, standardized pre-operative reports of the imaging of the venous system, both from Doppler-ultrasound examination and venography (either using magnetic resonance (MR) or computed tomography (CT)), medical reports from the embolization procedure including any medication or adverse events, as well as pre- and post-operative surveys with the assessment of symptoms intensity change and patients’ satisfaction scores.

The embolization procedure was conducted using embolization coils—Nester (Cook Medical, Bloomington, IN, USA) and/or Concerto (Medtronic/Micro Therapeutics Inc., Irvine, CA, USA) with the assistance of Zenition 70, X-ray/fluoroscopy C-arm device (Philips Medical Systems, Best, The Netherlands). The supplementary transcatheter foam sclerotherapy of the distal segments or branches of treated axes, as well as periuterine, paraovarian, paravaginal varices, and venous plexuses, was performed using polidocanol/lauromacrogol 400 (Aethoxysclerol, Kreussler Pharma, Wiesbaden, Germany) or sodium tetradecyl sulfate (Fibrovein, STD Pharmaceutical Products Ltd., Hereford, UK).

According to the routine protocol of the clinic, in some patients, further treatment for insufficient superficial veins in the lower limb compartment, was done if required. It included laser thermoablation of incompetent trunks and/or their large branches and, when necessary, it was supplemented by miniflebectomy or foam sclerotherapy of tributaries [6].

The main symptoms concerning pelvic and leg symptoms, and the abdominal pain during and after the embolization procedure were evaluated using a 0–10 points visual analogue scale (VAS). The overall treatment results and main symptom intensity change were self-assessed by each patient, at least 3 months after the last procedure, using the 4-grade scale: “(1) significant improvement”, “(2) moderate improvement”, “(3) no change” and “(4) worsening”.

The Shapiro-Wilk test was used to test the normal distribution of analyzed variables with a significance level *p* < 0.05. The baseline characteristics of the patient group, including age, body mass index, some parameters from the embolization procedure with the absorbed X-ray dose, the volume of sclerotizing foam, and VAS assessments, were analyzed using descriptive statistics involving the calculation of the arithmetic mean, median and standard deviations, which were then compared between respective groups using an unpaired Student’s *t*-test. The variables concerning the occurrence of main vascular abnormalities, previous treatment, or distribution of patient satisfaction scores, were shown as respective ratios or percentages. The latter were then compared between groups using Pearson’s chi-squared test, except for calculations concerning the local complications in the access site, where Fisher’s exact test appeared more appropriate due to the small sample size [18]. The impact of previous treatment on the occurrence of specified events was evaluated using a relative risk (RR) calculation, with a 95% confidence interval (95% CI). The patients’ self-assessment scores were also compared among groups using the Mann-Whitney U test. The observed differences were considered statistically significant at *p* < 0.05. All calculations were performed using the MedCalc Software 18.11v (www.medcalc.org) accessed in April–June 2024.

## 3. Results

All 506 records, each corresponding to a different individual, were selected for analysis and extracted from the database. The records conformed to the previously specified inclusion criteria and none of them fulfilled any of the exclusion criteria. According to patients’ history, more than one-third (*n* = 188; 37.2%) of them presented recurrent venous disease (ReVD), i.e., already underwent some venous treatment (surgery, thermoablation, non-thermal glue-mediated venous ablation, or sclerotherapy) in the lower limb compartment. Noteworthy, 58 patients from that subgroup (30.9%) have experienced three or more interventions in the past, nearly the same percentage (28.7%) was subjected to the venous procedure twice, whereas 76 individuals (40.4%) were treated only once. The mean age and body mass index of ReVD patients were slightly higher than those of non-treated patients (hereinafter referred to as the Intact group), nevertheless, these differences were non-significant. Interestingly, the comparison of mean symptom scores assessed before the treatment (baseline) revealed statistically significant differences between both groups. The abdominal and/or pelvic pain or discomfort was significantly higher in the Intact group, whereas the mean leg symptoms—pain/heaviness and visual scores were significantly higher (worse) in patients from the ReVD group (Table 1).

According to data from the MR- or CT-venography, the distribution of main vascular abnormalities in regard to the two predominant axes did not differ between groups. Significant reflux in the left ovarian vein (LOV) was found in 477 cases (94% of the whole group, with similar occurrence in both groups), whereas the right ovarian vein (ROV) was affected in nearly 70% of patients from both groups. Among patients from the ReVD group, 28 women (14.9%) revealed hypoplasia or significant compression of the left renal vein (LRV), which required high intraluminal pressure balloon venoplasty. Interestingly, in the Intact group, the LRV outflow obstruction, which was qualified for the venoplasty procedure, was detected in nearly one-third of patients (*n* = 101; 31.8%) and was significantly more frequent than in the ReVD group (Table 1).

The assessment of medical reports from EMBO of pelvic veins revealed that, although similar in both groups, the details of this procedure slightly differed between Intact and ReVD patients.

The transcutaneous endovascular pelvic vein embolization was performed using brachial and/or inguinal access (through the right common femoral vein or the proximal part of the right great saphenous vein). While the brachial access was used predominantly and with the same frequency (approximately 86%) in both groups, the inguinal access, which was necessary among others for balloon venoplasty, was used more frequently in the Intact than in the ReVD group and this difference was statistically significant (62.3% vs. 49.5%; at *p* = 0.005). As a consequence, the occurrence of main complications associated with the access site, hematoma/bruising and local inflammation/infection, also differed. The frequency of hematoma/bruising was similar in both groups and for both access sites. The occurrence of local inflammation/infection in brachial access was very low and significantly lower than in the case of inguinal access (0.7% vs. 4.8%; at *p* = 0.0005). When compared between groups, although the latter was higher in the Intact than in the ReVD group, this difference was non-significant (Table 2).

No serious complications, either technique-dependent (including clinically relevant venous wall dissection, significant blood loss and/or large extraperitoneal hematoma, coil migration, or pulmonary thromboembolism), or drug-related (e.g., severe anaphylaxis) were observed in any of treated individuals. Mild allergic reactions either systemic or local (skin erythema, rash, or itching) occurred in 27 individuals (5.4% of the whole group). Although slightly more frequent in the Intact group, they did not differ significantly when compared to the ReVD (Table 2).

**Figure 1:**
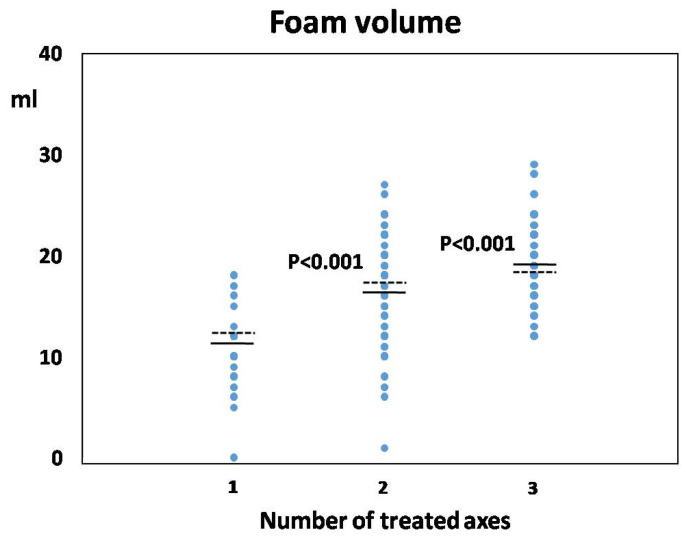
The volume of sclerotizing foam (in mL) in relation to the number of treated axes. Each dot represents data of a single patient, solid horizontal lines correspond to the mean foam volume used in the EMBO procedure for the respective number of treated axes, whereas dashed lines represent the median for this sub-group. *p* values indicate the differences between groups being statistically significant.

Approximately 60% of individuals from the whole group (*n* = 311) underwent treatment of two axes, one-fifth among them with LRV venoplasty (*n* = 67; 21.5%). Nearly 30% of all patients (*n* = 159) required treatment of only one axis (mainly LOV) and one-third among them (*n* = 58; 36.5%) with LRV venoplasty. Three axes were treated in 36 patients (7%), but only four individuals from the whole group (0.8%) required treatment of three axes with concomitant LRV venoplasty. Noteworthy, the overall distribution of treated axes did not differ between the Intact and ReVD groups; however, when comparing the frequency of LRV venoplasty for each number of treated venous axes, it was significantly higher in the Intact group (Table 2). As could be expected, the increasing number of treated axes was associated with a significantly higher volume of sclerotizing foam applied during the embolization procedure (Figure 1), although this relation did not differ between Intact and ReVD groups.

**Figure 2:**
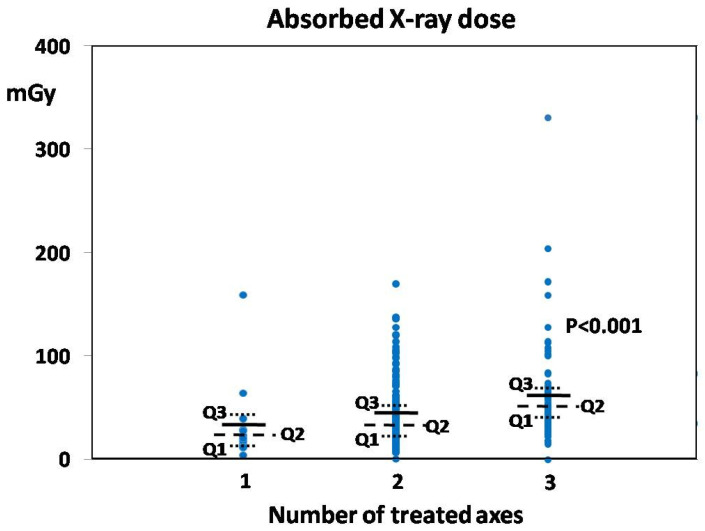
The dose of absorbed X-ray ionizing radiation (in mGy) in relation to the number of treated axes. Each dot represents data of a single patient, solid horizontal lines correspond to the mean absorbed dose applied during the EMBO procedure for the respective number of treated axes, dashed lines represent the median (second quartile—Q2), whereas dotted lines—respectively, first (Q1) and third (Q3) quartiles for this sub-group. The *p*-value indicates the difference between groups being statistically significant.

A similar association, although less pronounced, was observed between the number of treated axes and the absorbed dose of X-ray ionizing radiation expressed in milligrays (mGy). Although the mean absorbed dose gradually increased with the number of axes, it reached statistical significance only in patients who received treatment of three axes (Figure 2). Noteworthy, the mean and median values of the absorbed doses, when analyzed separately in patients who underwent embolization with LRV venoplasty, were slightly higher compared to respective values from embolization without venoplasty. However, due to the relatively large spread of those variables within each sub-group, those differences were non-significant. Again, no significant differences between Intact and ReVD groups were observed (Table 2).

Since general anesthesia was not used, the mild sedation for the EMBO procedure did not differ between both groups and involved benzodiazepine (midazolam), fentanyl, propofol, or their combinations. Four patients from the whole group (0.8%) did not require any medication at all. The types of medication and corresponding mean pain scores in the whole group were summarized in Table 3.

The pain experienced by the treated patients during the EMBO procedure was rather mild and in 433 individuals (85.5% of the whole group) it did not exceed 4 points in the 10-point VAS scoring scale. Moreover, 171 individuals (33.8% of the whole group) reported no pain at all (VAS = 0), whereas only 30 (5.9%) of them assessed the pain as nagging, with VAS ≥ 7. Noteworthy, within the first week after embolization, the mean pain score, although significantly higher when compared to that during the procedure, was still relatively low (VAS = 2.6 vs. 2.0), with 89 patients (17.6% of all individuals) reporting no pain even without any medication.

When compared between groups, the mean pain during and within the first week after the procedure, although relatively low, was significantly higher in the Intact group (Table 2). Interestingly, 79 individuals (42.0%) from the ReVD group reported no pain during EMBO and their percentage was significantly higher compared to the 28.9% (*n* = 92) from the Intact group. Within the first week after the embolization procedure, the mean pain increased in both groups. Consequently, the percentages of individuals reporting no pain decreased significantly, but they were still higher in the ReVD than in the Intact group (23.4% vs. 14.2%, respectively).

**Figure 3:**
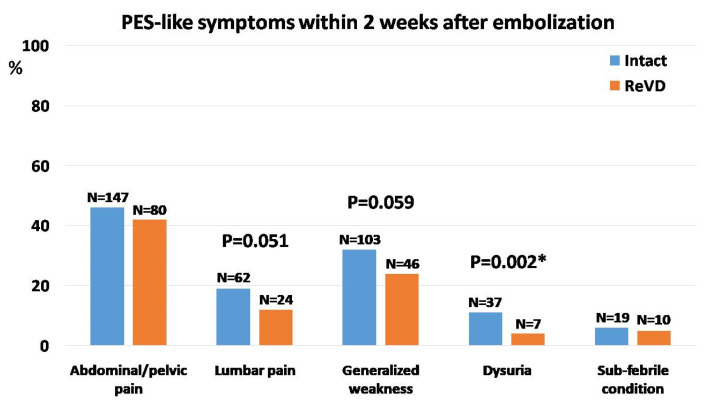
The distribution of main symptoms typical for post-embolization syndrome (PES) within 2 weeks after the embolization procedure. The bars represent percentages (with numbers) of individuals within each group reporting respective symptoms; differences between groups marked by *p* values with asterisks (*) were statistically significant.

In further observations the mild pelvic/abdominal pain or discomfort still persisted in nearly 45% of patients (*n* = 227) from the whole group for at least two weeks after embolization; however, its frequency or intensity did not differ between groups. Also, the occurrence of other symptoms typical for post-embolization syndrome (PES) was observed within 2 weeks after the procedure in 58.5% of patients (*n* = 296) from the whole group. Although slightly more frequent in the Intact than in the ReVD group, they did not differ significantly, except for dysuric symptoms. Apart from the aforementioned abdominal/pelvic pain, the other most common PES-like symptoms were generalized weakness and lumbar pain, which were reported, respectively, in 149 (29.4%) and 86 (17.0%) patients from the whole group (Table 2 and Figure 3).

Noteworthy, in the whole group the lumbar pain occurred in 32 patients (24.8%) who underwent LRV venoplasty (*n* = 129), but in only 54 of the patients (14.3%) without venoplasty (*n* = 377), and this difference was statistically significant. The relative risk of lumbar pain after LRV venoplasty for the whole group was calculated as 1.7-fold higher than without it (RR = 1.73; with 95% CI 1.17–2.55; at *p* = 0.006). Interestingly, when analyzed for each group separately, the occurrence of lumbar pain was reported by 5 patients (17.8%) after LRV venoplasty (*n* = 28) in the ReVD group, in contrast to 27 patients (26.7%) who underwent venoplasty in the Intact group (*n* = 101). Furthermore, the relative risk of lumbar pain after venoplasty in the ReVD group was lower and non-significant (RR = 1.50; with 95% CI 0.61–3.69; at *p* = 0.37), whereas in the Intact group, it was slightly higher and reached statistical significance (RR = 1.66; with 95% CI 1.06–2.58; at *p* = 0.02). No particular treatment for this condition was used except for occasional upon-request application of non-steroid anti-inflammatory drugs—ibuprofen or nimesulide.

Since nearly two-thirds of patients (*n* = 321; 63.4%) after abdominal and pelvic vein embolization required further venous treatment in the lower limb compartment (classified as C2 according to CEAP classification), data from surveys concerning symptom changes and overall satisfaction were assessed in two steps. At first, data were grouped and compared in regard to treatment protocol: embolization only (EMBO) versus embolization with further leg treatment (EMBO+LEGS). Data were subsequently analyzed within each treatment mode group in regard to previous treatment history (Intact vs. ReVD).

**Figure 4:**
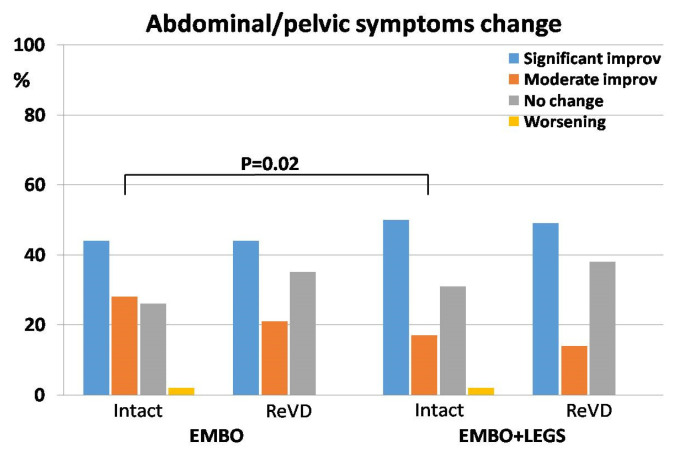
The distribution of patients’ survey scores—the change of abdominal or pelvic symptoms after respective treatment protocol (EMBO—patients subjected to embolization only; EMBO+LEGS—patients subjected to full sequential treatment) and in regard to the previous treatment history (Intact—no previous treatment, ReVD—recurrent venous disease). *p* = 0.02—difference between occurrence of moderate improvement reported by Intact patients in regard to the treatment protocol.

The assessment of pelvic/abdominal symptom change surveys has shown that 337 patients (66.6%) from the whole group experienced overall improvement, which 47.2% of them (*n* = 239) assessed as “significant”. Nearly one-third (*n* = 162; 32.0%) of patients declared “no change” whereas 1.4% of them (*n* = 7) reported “worsening”. Similar results were obtained when data were analyzed in regard to the treatment protocol, except for the lower percentage of patients who declared moderate improvement in the EMBO+LEGS group, especially in the Intact subgroup, which was significantly lower, compared to the Intact subgroup with the EMBO protocol, i.e., 17.1% vs. 27.7%, respectively (Figure 4 and Table 4).

**Figure 5:**
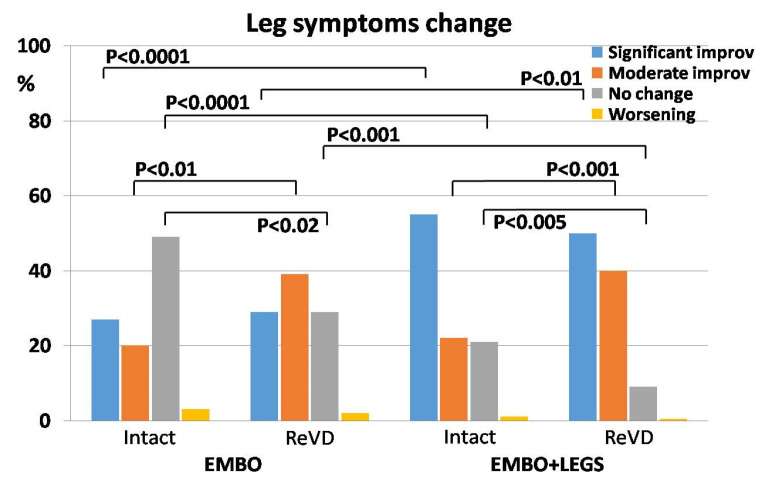
The distribution of patients’ survey scores—the change of leg symptoms after respective treatment protocol (EMBO—patients subjected to embolization only; EMBO+LEGS—patients subjected to full sequential treatment) and in regard to the previous treatment history (Intact—no previous treatment, ReVD—recurrent venous disease). *p* values indicate the differences between respective groups being statistically significant.

The analysis of leg symptom change surveys revealed an improvement in 365 (72.1%) of all treated patients, which was assessed as “significant” in 221 (43.7%) of them. No symptom changes or worsening estimates were declared by 133 (26.3%) and 8 (1.6%) of all patients, respectively. When analyzed in regard to the treatment protocol, the “significant improvement” was declared more frequently in the EMBO+LEGS group, compared to EMBO alone (53.0% vs. 27.6%, respectively) (Table 4 and Figure 5).

Notably, the occurrence of patients who declared moderate improvement significantly differed between the Intact and ReVD subgroups, although this difference was similar within the EMBO and EMBO+LEGS treatment protocol groups.

Nearly half of the Intact subgroup from the EMBO protocol declared “no change” in the leg symptom survey and it differed significantly from all other groups. The lowest occurrence of “no change” (9.3%) was reported in the ReVD subgroup with EMBO+LEGS protocol. It differed significantly from the Intact subgroup with the same treatment protocol, as well as from the respective ReVD subgroup with the EMBO protocol (Table 4 and Figure 5).

**Figure 6:**
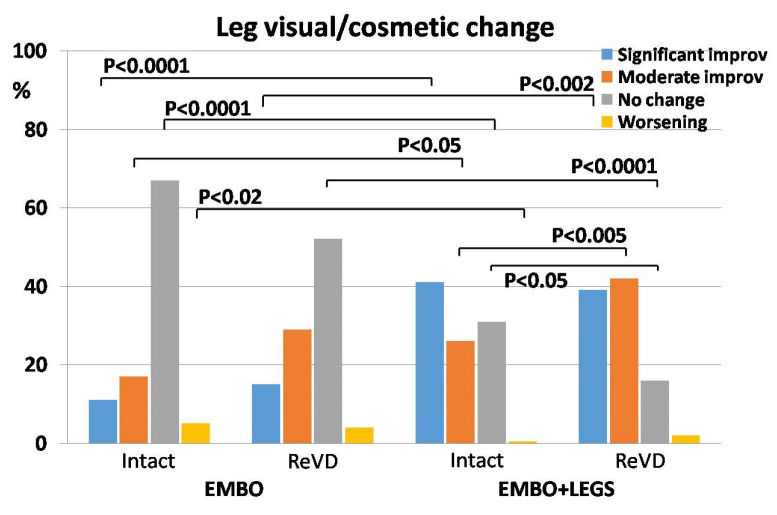
The distribution of patients’ survey scores—the change of leg visual/cosmetic aspects after respective treatment protocol (EMBO—patients subjected to embolization only; EMBO+LEGS—patients subjected to full sequential treatment) and in regard to the previous treatment history (Intact—no previous treatment, ReVD—recurrent venous disease). *p* values indicate the differences between respective groups being statistically significant.

The most differentiated results were obtained in surveys concerning leg visual/cosmetic changes. The improvement was reported by 296 (58.5%) of all patients, but only half of them (*n* = 152; 30.0%) assessed it as “significant”. The occurrence of “no change” and “worsening” was declared, respectively, by 203 (40.1%) and 13 (2.6%) of all patients. When analyzed in regard to the treatment protocol, the occurrence of patients who declared significant improvement was four-fold higher in the EMBO+LEGS protocol compared to EMBO alone. Consequently, it resulted in a marked shift to “no change” estimates, which occurred most frequently in the EMBO protocol, especially in the Intact subgroup, but they were the rarest in the ReVD subgroup with the EMBO+LEGS protocol (67.1% vs. 16.5%, respectively). Notably, in this survey, the highest occurrence of “worsening” was reported by patients from the Intact subgroup with the EMBO protocol which was significantly higher compared to the respective Intact subgroup with EMBO+LEGS (5.1% vs. 0.6%, respectively) (Table 4 and Figure 6).

## 4. Discussion

The embolization of pelvic veins was proposed for the first time as a treatment method thirty years ago by Edwards et al. [19]; nevertheless, its position in the complex treatment of venous disease is relatively new and still lacking strong scientific evidence [4,20]. Therefore, in recent Clinical Practice Guidelines on the Management of Chronic Venous Disease of the Lower Limbs [10], PVI treatment received the recommendation class 2a—i.e., “weight of evidence/opinion is in favour of usefulness/efficacy”. On the other hand, the same document contains the recommendation of class 3 (i.e., “evidence or general agreement that the given treatment or procedure is not useful/effective, and in some cases may be harmful”), which states: “No pelvic veins embolization in patients with varicose veins of pelvic origin without pelvic symptoms” [10]. Actually, this point of view is extremely surprising, as it neglects numerous observations concerning the involvement of PVI in recurrence after previous leg treatment, but also completely ignores the compensatory role of the lower limb compartment in the reduction of pelvic symptoms [6,9]. Due to the large variety of the latter, their occurrence is usually underestimated and some of them are omitted by patients or clinicians, possibly because women are embarrassed to talk about them. As a matter of fact, the proper symptom evaluation requires adequate tools, which wouldn’t focus on either pelvic or leg symptoms only [20,21,22,23]. Such tools should concern the overall disease-specific, health-related quality of life. Moreover, they should assess the entire physical, psychological, and social burden of venous disease on women, and not just the effects of the pain. However, so far, such tools are not available and still need to be developed.

Therefore, there is a strong demand to provide more data necessary to confirm the rationale for the EMBO procedure in sequential treatment of venous disease, especially in patients with low levels of PVI symptoms [6,22,23]. Our current report may be very useful in that matter as it concerns the largest, to our best knowledge, homogeneous data set from sequential treatment, which was conducted in a single center. Also, since it focuses on the safety and short-term efficacy of the sequential approach, it may provide valuable insight into the assessment of its benefit-risk ratio.

Since the use of the EMBO procedure in PVI treatment has a thirty-year history, the safety of this method is relatively well studied [2,14]. Nevertheless, the introduction of fluoroscopy and the use of the digital subtraction method in intraoperative imaging allowed a significant reduction of patient exposure to X-ray radiation during the EMBO procedure. Furthermore, the technological development of CT and MR devices enabled better preoperative diagnostics with more detailed imaging of pathology qualified for treatment. However, despite some attempts to use artificial intelligence in the assessment of CT or MR scans [24,25], the opinion of an experienced clinician remains the most critical factor for proper patient qualification and procedure planning. The well-planned endovascular intervention performed by a qualified and trained team is relatively short (usually below 60 min), which enables a significant reduction of X-ray exposure and thus improves the safety of the EMBO procedure in the treatment of PVI. In our study, the key factor that influenced the patient’s exposure to radiation was the number of axes being treated. The embolization procedure of three axes, when compared to one axis only, was longer and required more intraoperative X-ray exposures, which consequently resulted in a higher absorbed dose. Nevertheless, the mean radiation dose absorbed by patients during EMBO in our study was nearly 9-fold lower compared to that reported by some other authors (43 mGy vs. 398 mGy) [26]. Moreover, the mean absorbed dose mentioned was similar to or even lower than that received during CT examination or some single X-ray shots (e.g., used for assessment of pelvic bones or vertebral column in orthopedics and traumatology) [27].

Other worries associated with the EMBO procedure are possible technique-related complications [14,22]. Although even with highly experienced professionals any serious adverse events cannot be entirely excluded; the risk of their occurrence is very low and may be further decreased using some technical tips and tricks. One of them is the ultrasound-assisted venous puncture to reduce local vessel damage and avoid severe bleeding in the access site. In our study, this approach allowed us to elude the risk of a large local hematoma, whereas small bruising occurred in less than 15% of individuals, independently of the access site. The higher frequency of inflammatory/infectious reaction in inguinal access was due to the less comfortable and more demanding maintenance of the hygiene of the groin compared to brachial access. The risk of allergic reactions to the contrasting agent may be decreased by its dilution and the use of a high-quality imaging system. In our group, no severe anaphylaxis was observed, whereas mild allergic reactions occurred in 5% of patients and mainly affected the skin.

Other serious complications, including clinically relevant venous wall dissection, significant blood loss, coil migration, pulmonary thromboembolism, pneumothorax, or severe cardiac arrhythmias (which may occur with brachial or jugular access when guidewire or catheter is passing through the right heart atrium), although reported by several authors, were not observed in our study [4,14,23].

The mean pain score reported by patients during the EMBO procedure was low, but one has to keep in mind that, actually, in a vast majority of patients, except for 0.8% from the whole group, it was controlled pharmacologically. Therefore, although the mean pain score within the first week after EMBO was significantly higher than during that procedure, it was still relatively low and gradually decreased. Within the next week (2 weeks after EMBO) no symptoms, including pain, were reported by 41.5% of patients from the whole group.

The occurrence of pain during and after the EMBO procedure, especially that located in the lumbar region, could be explained, at least in some patients, by LRV venoplasty. Indeed, we found that this intervention increased the risk of lumbar pain 1.7-fold in the whole group. Notably, this increase was statistically significant in the Intact, but not in the ReVD group, presumably due to the lower frequency of LRV venoplasty in the latter.

The typical signs for post-embolization syndrome (PES-like), although being defined as those emerging within 72 h after embolization, were reported by our patients even up to two weeks after the EMBO procedure. Interestingly, their frequency was significantly higher when compared to that reported by other authors (up to 60% vs. 20%) [2,4,14,23]. Nevertheless, in our study, the symptom intensity was rather low, except for a few cases with moderate pelvic pain/discomfort and patients did not require any particular medication.

Taken together, the assessment of the benefit-risk balance for the embolization procedure clearly shows its high efficacy in the short-term follow-up after the sequential treatment of venous disease. The safety issues require and may sufficiently be managed by both thorough preoperative imaging and careful procedure planning, which significantly reduce radiation exposure and minimize the procedural risks. Hence, the intervention is associated with a negligible risk of serious complications, especially when performed by an experienced team and after detailed diagnostics. However, the mid- and long-term observations are still lacking and need to be continued.

The secondary goal of our study was the assessment and explanation of the possible impact of previous leg treatment on the efficacy of the EMBO procedure alone or in combination with further intervention in the lower limb compartment. The rationale for such analysis was the observation that after sequential treatment, patients with ReVD usually experience better improvement than those without any previous interventions.

Here we propose a possible explanation of that phenomenon. As shown in our previous study [9], 85.6% of patients with ReVD revealed underdiagnosed or omitted PVI, which concerned practically all patients who underwent LLVI treatment more than 10 years ago. Furthermore, in nearly 30% of these cases, we found various anatomical variations or abnormalities, mainly affecting the LRV-LOV axis. In such patients, the intervention in the leg compartment only, predominantly careless high ligation and stripping, significantly affects the hemodynamics of the venous system in the groin and in the pelvic floor.

Without the elimination of the pelvic source of reflux, the clinically overt recurrence in the lower limb compartment in the majority of patients usually emerges in less than five years [9]. Therefore, this group may be used as a good model for studying the influence of treatment on symptom distribution—in ReVD patients shortly after leg treatment, similar to intact with PVI only, pelvic symptoms should predominate [3,22,23]. When leg veins deteriorate, they may relieve or compensate for pelvic vein overload. The pelvic symptoms could alleviate and move downward, to the lower limb compartment, where they become more annoying, including cosmetic worsening, too. Although disease progression in the leg compartment may concern both intact and ReVD patients, in the latter, due to aforementioned postoperative hemodynamics alterations, mean leg symptoms could be significantly worse. However, after reducing pelvic vein overload through EMBO and performing hemodynamic correction of leg veins patients who initially reported higher symptom intensity should experience the most significant improvement in the lower limb compartment, in regard to both symptoms and visual aspects.

Interestingly, besides the increased difficulty of endovenous treatment after high ligation and stripping, the type of previous interventions did not have any significant impact on the final outcome of the sequential treatment.

In accordance with our hypothesis, there was no significant difference between groups in regard to pelvic symptoms after treatment—the significant or moderate improvement was similar in both groups, whereas no change in pelvic symptoms, but with simultaneous improvement in leg symptoms, was reported mainly by individuals with a low baseline score of pelvic pain.

Furthermore, as could be expected in regard to leg symptoms, including visual/cosmetic changes, the majority of ReVD patients, especially those after the EMBO+LEGS protocol, experienced significant or at least moderate improvement, whereas the lowest occurrence of “improvement” estimates was reported in the Intact/EMBO subgroup. On the other hand, the latter most frequently reported “no change”. However, this finding should not be surprising as the majority of those patients did not require or undergo any supplementary treatment in the leg compartment and had no symptoms at the beginning; therefore, they actually did not experience any improvement and truly reported “no change”.

Finally, in the same group (Intact/EMBO) we noted the highest occurrence of “worsening” estimates, especially in regard to leg visual/cosmetic changes. We have no clear explanation for this finding. Possibly, with relatively low other symptoms, these patients were mainly focused on their higher esthetic expectations [6]. In fact, based on available visit reports, the only abnormalities in the lower limb compartment remaining after the EMBO procedure in these patients were small telangiectasias and reticular veins on the lateral aspect of the thigh, which apparently affected the overall patients’ satisfaction.

The main limitations of this study are its retrospective nature and the specific patient selection, which does not necessarily conform with the distribution of the aforementioned pathology in the whole population. The other weak point is the very short period, which needs to be extended to the short- and mid-term observation (in preparation).

## 5. Conclusions

Our study provides the largest homogeneous dataset from a single-center sequential treatment of venous disease to date. Despite its retrospective nature and specific patient selection, which may not reflect the whole population, it offers valuable insights into the safety and short-term efficacy of the embolization procedure in sequential treatment, especially in patients with recurrence.

Despite some skepticism in the medical community regarding the sequential treatment of pelvic and lower limb venous compartments, our findings support the benefit of this approach, where addressing PVI may alleviate disease symptoms and has a significant impact on the further management of the lower limb superficial veins. The main controversy concerns the proper qualification for treatment, particularly in patients with low intensity of PVI symptoms, which can easily be overlooked due to patient embarrassment or inadequate diagnostic tools. Therefore, we emphasize the need for further research, including randomized controlled trials, to develop standardized, effective treatment protocols and solidify the role of pelvic vein embolization in the comprehensive management of venous disease.

## Figures and Tables

**Table 1:** Baseline characteristics of the groups.

Feature/Parameter	Whole Group (*n* = 506)	Intact Group (*n* = 318)	ReVD Group (*n* = 188)
Age (mean/median; ±SD)	40.6/39.0; ±9.0	38.5/38.0; ±8.4	44.3/42.0; ±8.9
BMI (mean/median; ±SD)	22.9/22.3; ±3.7	22.5/22.1; ±3.5	23.4/22.7; ±4.0
Symptoms at baseline (mean/median; ±SD)			
Abdominal/pelvic pain (VAS 0–10)	4.3/4.0; ±3.3	4.9/5.0; ±3.3 *	3.4/3.0; ±3.2 *
Leg pain/discomfort (VAS 0–10)	5.1/5.0; ±2.9	4.7/5.0; ±3.0 *	5.7/6.0; ±2.7 *
Leg visual/cosmetic aspects (VAS 0–10)	5.1/5.0; ±3.1	4.6/5.0; ±3.2 *	5.9/6.0; ±2.7 *
CEAP classification (*n*; %)			
C1	185 (36.6%)	137 (43.1%) *	48 (25.5%) *
C2	321 (63.4%)	181 (56.9%) *	140 (74.5%) *
Affected abdominal/pelvic vessels (*n*; %)			
LOV	477 (94.3%)	300 (94.3%)	177 (94.1%)
ROV	345 (68.2%)	215 (67.6%)	130 (69.1%)
LIILV	28 (5.5%)	13 (4.1%)	15 (8.0%)
RIILV	38 (7.5%)	17 (5.3%) *	21 (11.2%) *
LRV	129 (25.5%)	101 (31.8%) *	28 (14.9%) *
Retroperitoneal collaterals	27 (5.3%)	18 (5.7%)	9 (4.8%)

Table legend: BMI—body mass index; CEAP—clinical, etiology, anatomy and pathophysiology classification; VAS—visual analogue scale; LOV—left ovarian vein; ROV—right ovarian vein; LIILV—left internal iliac vein; RIILV—right internal iliac vein; LRV—left renal vein; * statistically significant difference between groups, at *p* < 0.05.

**Table 2:** Main features and adverse events associated with embolization procedure.

Feature/Parameter	Whole Group (*n* = 506)	Intact Group (*n* = 318)	ReVD Group (*n* = 188)
**Access site (*n*; %)**			
Brachial access	437 (86.4%)	271 (85.2%)	166 (88.3%)
Hematoma/bruising in access site	56 (12.8%) ^1^	39 (14.4%) ^1^	17 (10.2%) ^1^
Inflammation/infection in access site	3 (0.7%) ^1^	2 (0.7%) ^1^	1 (0.6%) ^1^
Inguinal access	291 (57.5%)	198 (62.3%) *	93 (49.5%) *
Hematoma/bruising in access site	38 (13.1%) ^2^	26 (13.1%) ^2^	12 (12.9%) ^2^
Inflammation/infection in access site	14 (4.8%) ^2^	12 (6.1%) ^2^	2 (2.1%) ^2^
**Number of treated axes (*n*; %)**			
1	101 (20.0%)	59 (18.6%)	42 (22.3%)
1+LRV venoplasty	58 (11.5%)	48 (15.1%) *	10 (5.3%) *
2	244 (48.2%)	143 (45.0%)	101 (53.7%)
2+LRV venoplasty	67 (13.2%)	51 (16.0%) *	16 (8.5%) *
3	32 (6.3%)	15 (4.7%)	17 (9.0%)
3+LRV venoplasty	4 (0.8%)	2 (0.6%)	2 (1.1%)
X-ray absorbed dose in mGy (mean/median; ±SD)	43.2/33.9; ±35.7	40.0/33.7; ±27.7	48.5/34.3; ±45.4
Foam volume in ml (mean/median; ±SD)	16.4/17.0; ±4.7	16.2/17.0; ±4.6	16.7/18.0; ±4.9
Systemic allergic reaction (n; %)	10 (2%)	8 (2.5%)	2 (1.1%)
Skin erythema/rash/itching (n; %)	17 (3.4%)	12 (3.8%)	5 (2.7%)
**Pain score (VAS 0–10)**			
during procedure (mean/median; ±SD)	2.0/1.0; ±2.2 #	2.2/2.0; ±2.3 *#	1.6/1.0; ±1.9 *#
within 1 week (mean/median; ±SD)	2.6/2.0; ±2.2 #	2.9/3.0; ±2.2 *#	2.1/2.0; ±1.9 *#
**PES-like symptoms (*n*; %)**			
Abdominal/pelvic pain	227 (44.9%)	147 (46.2%)	80 (42.6%)
Lumbar pain	86 (17.0%)	62 (19.5%)	24 (12.8%)
Dysuria	44 (8.7%)	37 (11.6%) *	7 (3.7%) *
Generalized weakness	149 (29.4%)	103 (32.4%)	46 (24.5%)
Sub-febrile condition	29 (5.7%)	19 (6.0%)	10 (5.3%)
No symptoms	210 (41.5%)	123 (38.7%)	87 (46.3%)

Table legend: ^1,2^—frequencies calculated in regard to respective access site; * difference statistically significant between groups, at *p* < 0.05; # difference statistically significant within-group (compared between pain during and 1 week after procedure), at *p* < 0.05; PES—post-embolization syndrome.

**Table 3:** The distribution of respective types of medication and mean pain scores during the EMBO procedure.

Type of Anesthesia	Number of Individuals (*n*; %)	Pain Score (VAS 0–10)	
		Mean/Median; ±SD	Quartile Q1; Q2; Q3
No anesthesia	4 (0.8%)	1.5/2.0; ±1.0	1.5; 2.0; 2.0
Benzodiazepine (BDZ)	389 (76.9%)	2.0/1.0; ±2.2	0.0; 1.0; 3.0
Fentanyl (FENT)	41 (8.1%)	2.8/2.0; ±2.4	1.0; 2.0; 4.0
Propofol (PROP)	0	-	-
BDZ and FENT	65 (12.8%)	1.6/1.0; ±2.1	0.0; 1.0; 2.0
BDZ and PROP	6 (1.2%)	1.7/2.0; ±1.0	1.25; 2.0; 2.0
BDZ and FENT and PROP	1 (0.2%)	1.0	1.0

**Table 4:** The distribution of symptom change scores assessed in follow-up surveys.

Evaluation of Symptom Change	EMBO (*n* = 185; 36.6%)		EMBO+LEGS (*n* = 321; 63.4%)	
	Intact (*n* = 137; 74.1%)	ReVD (*n* = 48; 25.9%)	Intact (*n* = 181; 56.4%)	ReVD (*n* = 140; 43.6%)
**Abdominal/pelvic symptoms**				
Significant improvement	60 (43.8%)	21 (43.8%)	90 (49.7%)	68 (48.6%)
Moderate improvement	38 (27.7%) *	10 (20.8%)	31 (17.1%) *	19 (13.6%)
No change	36 (26.3%)	17 (35.4%)	56 (31.0%)	53 (37.8%)
Worsening	3 (2.2%)	0 (0.0%)	4 (2.2%)	0 (0.0%)
**Leg symptoms change**				
Significant improvement	37 (27.0%) *	14 (29.2%) **	100 (55.2%) *	70 (50.0%) **
Moderate improvement	28 (20.4%) #	19 (39.6%) #	41 (22.6%) ##	56 (40.0%) ##
No change	68 (49.6%) *#	14 (29.2%) **#	38 (21.0%) *##	13 (9.3%) **##
Worsening	4 (2.9%)	1 (2.1%)	2 (1.1%)	1 (0.7%)
**Leg visual/cosmetic change**				
Significant improvement	15 (10.9%) *	7 (14.6%) **	75 (41.4%) *	55 (39.3%) **
Moderate improvement	23 (16.8%) *	14 (29.2%)	48 (26.5%) *#	59 (42.1%) #
No change	92 (67.1%) *	25 (52.1%) **	57 (31.5%) *#	29 (16.5%) **#
Worsening	7 (5.1%) *	2 (4.2%)	1 (0.6%) *	3 (2.1%)

Table legend: EMBO—patients subjected to embolization only; EMBO+LEGS—patients subjected to the full sequential treatment (embolization first, the intervention in the lower limb compartment next); *, **—difference between respective protocol groups (EMBO vs. EMBO+LEGS); #, ##—the difference within respective protocol group (Intact vs. ReVD).

## Data Availability

Due to law restrictions aimed to protect patient confidentiality, the original data are not publicly available; however, the data presented in this report are available from the corresponding author upon request.
